# Severe weather events and cryptosporidiosis in Aotearoa New Zealand: A case series of space–time clusters

**DOI:** 10.1017/S095026882400058X

**Published:** 2024-04-15

**Authors:** Leah Grout, Simon Hales, Michael G. Baker, Nigel French, Nick Wilson

**Affiliations:** 1 Department of Public Health, University of Otago, Wellington, New Zealand; 2 Tāwharau Ora, School of Veterinary Science, Massey University, Palmerston North, New Zealand

**Keywords:** storms, severe weather events, flooding, infectious disease, *Cryptosporidium* spp, surveillance, New Zealand

## Abstract

Occurrence of cryptosporidiosis has been associated with weather conditions in many settings internationally. We explored statistical clusters of human cryptosporidiosis and their relationship with severe weather events in New Zealand (NZ). Notified cases of cryptosporidiosis from 1997 to 2015 were obtained from the national surveillance system. Retrospective space–time permutation was used to identify statistical clusters. Cluster data were compared to severe weather events in a national database. SaTScan analysis detected 38 statistically significant cryptosporidiosis clusters. Around a third (34.2%, 13/38) of these clusters showed temporal and spatial alignment with severe weather events. Of these, nearly half (46.2%, 6/13) occurred in the spring. Only five (38%, 5/13) of these clusters corresponded to a previously reported cryptosporidiosis outbreak. This study provides additional evidence that severe weather events may contribute to the development of some cryptosporidiosis clusters. Further research on this association is needed as rainfall intensity is projected to rise in NZ due to climate change. The findings also provide further arguments for upgrading the quality of drinking water sources to minimize contamination with pathogens from runoff from livestock agriculture.

## Introduction

Cryptosporidiosis incidence has been associated with weather and climatic conditions [1, 2]. In Aotearoa New Zealand (NZ), cryptosporidiosis has been associated with rainfall [3–5] and temperature [3, 4, 6]. Heavy rainfall events can increase surface runoff of cryptosporidium oocysts in the environment [7–10], leading to increased cryptosporidium pathogen loads in waterways [11–13]. *Cryptosporidium* spp. are resistant to conventional water treatment techniques [14], and increased pathogen loading due to heavy rainfall events can overwhelm potable water and wastewater infrastructure and lead to disease outbreaks [15]. Cryptosporidiosis cases are often linked to contaminated water [16–18], and *Cryptosporidium* spp. are one of the most reported causal agents for waterborne outbreaks of enteric disease in NZ [19]. Rainfall-associated runoff could exacerbate the risk related to the consumption of untreated drinking water, and higher cryptosporidiosis rates have been reported in areas with untreated or inadequate drinking water supplies in NZ [20]. Recreational water contact has also been identified as an important risk factor for infection [6].

Space–time cluster detection methods have previously been used to investigate the clustering of a number of infectious diseases including listeriosis in the USA [21], shigellosis in the USA [22], and cryptosporidiosis in NZ [23]. Specifically, the latter NZ study examined recurrent clusters of sporadic cryptosporidiosis cases for three time periods: 1997–2001, 2001–2004, and 2005–2008 using SaTScan software to identify locations and time periods of increased risk [23]. The study found that many of the statistically significant cryptosporidiosis clusters were in areas with high livestock land use and occurred during the spring [23]. The detection of space–time clusters of cryptosporidiosis may help to identify temporal and spatial risk factors, which could in turn inform disease prevention and control efforts. Therefore, this analysis aimed to (i) detect spatiotemporal foci in the incidence of cryptosporidiosis in NZ across a longer period (1995–2015), (ii) compare the timing and location of detected clusters to severe weather events recorded in the National Institute of Water and Atmospheric Research’s (NIWA) Historic Weather Event Catalogue, and (iii) compare detected clusters to reported outbreaks.

## Methods

### Data collection

All notified cases of cryptosporidiosis from 1997 to 2015 in NZ were obtained from the National Notifiable Disease Surveillance System, which is maintained by the Institute of Environmental Science and Research (ESR). The report date for cryptosporidiosis notifications was used for this study. Both sporadic cases, and cases identified as being part of known outbreaks, were included.

Census area unit (CAU; n = 1976) boundaries for the 2006 National Census and population data were obtained from Statistics NZ (the national official statistics agency). CAUs are non-administrative geographic units that vary in size, each with a population ranging from around 3,000 to 5,000 people. CAU centroids were calculated using ArcGIS Desktop v10.5.1 [24].

Daily weather data, including total precipitation (m) and maximum temperature (K), were obtained from the ERA-Interim database at the European Centre for Medium-Range Forecasts [25]. The weather estimates were originally provided at 0.25 degrees resolution but were summarized at the 2006 CAU level.

### Space–time cluster detection

Cryptosporidiosis clusters from 1997 to 2015 in NZ were identified using the Kulldorff method of retrospective space–time permutation [26] in SaTScan™ [27]. The analysis was carried out at the CAU level with aggregated, unadjusted count data. This method automatically adjusts for purely spatial patterns that are constant over time, as well as purely temporal trends, such as seasonal patterns of disease. The space–time scan statistic is defined by a cylindrical window with a circular geographic base and with height corresponding to time. As the cylindrical window moves across the study area, it scans the specified time period and highlights potential spatiotemporal clusters of disease cases for each location. The size of the cylindrical window was limited to a radius of 25 km and a maximum of 30 days in order to identify smaller clusters (i.e. at least three cases) that may indicate localized exposure.

Monte Carlo replication was used to explore the statistical significance of detected clusters. A *p-*value was obtained by ranking the likelihood of an observed cluster in a data set over the maximum likelihoods acquired by generating 999 randomly produced data sets. The null hypothesis of ‘no cluster’ was rejected when the simulated *p*-value was less than 0.05.

SaTScan required the geographic coordinates for the CAU centroids corresponding to each case, but population data were not required when using a space–time permutation model. SaTScan provides HyperText Markup Language (HTML) files for temporal graphs and maps of clusters, but the output from SaTScan was exported and statistically significant clusters were mapped in ArcGIS.

### Comparison to severe weather events

NIWA is the lead Crown Research Institute responsible for collecting data on climate and weather hazards in NZ. NIWA’s Historic Weather Events Catalogue (https://hwe.niwa.co.nz/) was searched for events with evidence of increased rainfall that occurred within the 21 days preceding each significant cryptosporidiosis cluster. A severe weather event can mobilize cryptosporidium oocysts in the environment through storm water runoff, and flooding can increase the contamination of surface waters during and after heavy rainfall [2, 13]. Cryptosporidium oocysts can retain viability and infectivity for more than 12 weeks in the environment [28], and while cryptosporidium oocyst numbers are typically reduced over time in water, they remain infective for the longest time in colder temperatures and can retain viability and infectivity after freezing [28–30]. In addition, the average incubation period for cryptosporidiosis has been reported to be around seven days [31–34], with a minimum incubation period of around one to two days [31–33]. Therefore, to account in part for high concentrations of cryptosporidium oocysts in surface waters following severe weather events that could retain viability and infectivity for longer periods, as well as the incubation period for cryptosporidiosis in humans, a period of 21 days (i.e. three times the average incubation period of seven days) was used to assess temporal alignment between severe weather events and significant clusters. Severe weather events also had to be reported to affect the region in which the cluster was detected to be considered spatially aligned. Alignment was also checked by exploring temporal patterns of weather data for the first listed CAU within each cluster (see ‘Comparison to daily total precipitation and maximum temperature’).

### Comparison to daily total precipitation and maximum temperature

For the significant cryptosporidiosis clusters that aligned with severe weather events, the temporal patterns of daily total precipitation and maximum temperature were plotted prior to and for the duration of the detected clusters. Specifically, the weather variables were plotted for the identifying CAU for each relevant cluster (i.e. if there were multiple CAUs within the cluster boundary, weather data were only plotted for the first CAU listed by SaTScan).

### Comparison to previously recorded outbreaks

For the statistically significant cryptosporidiosis clusters that aligned with severe weather events, if a case, or cases, within one of the detected clusters had been assigned an outbreak number, then information about the outbreak was requested from ESR for comparison to the cluster. Specifically, data on the timing, location, and number of cases were compared to the detected clusters and information on potential causes and modes of transmission was also examined. ESR defines the start date of an outbreak as the date of the onset of illness in the first reported case.

## Results

### Space–time cluster detection

A total of 15,822 cryptosporidiosis cases were notified in NZ from 1997 to 2015. SaTScan analysis detected 65 cryptosporidiosis clusters during the study period. A majority of those clusters (58.5%, 38/65) were statistically significant (*p* < 0.05). The statistically significant clusters were less likely to have occurred by chance than the clusters that were not statistically significant. In total, 645 (4.1%) notified cryptosporidiosis cases were identified by SaTScan as being part of significant clusters (Table 1). The number of cases for significant clusters ranged from 3 to 83 (mean = 17.0, median = 10).

**Table 1.** Statistically significant cryptosporidiosis space–time clusters (*p* < 0.05) identified by SaTScan in New Zealand, 1997–2015

Cluster number	Start date (yyyy/mm/dd)	End date (yyyy/mm/dd)	*P*-value	Observed cases (N)	Expected cases (N)
1	1998/03/26	1998/04/24	1.000E–17	83	4.6
2	1999/03/23	1999/04/20	1.000E–17	55	2.0
3	2002/07/24	2002/08/14	1.000E–17	32	0.6
4	2001/03/01	2001/03/30	1.000E–17	56	4.8
5	2006/10/06	2006/10/07	1.000E–17	23	0.5
6	2000/10/25	2000/10/27	1.000E–17	22	0.5
7	2001/03/26	2001/04/24	3.300E–16	65	13.6
8	1999/08/25	1999/09/22	1.692E–11	26	2.3
9	2010/01/07	2010/02/03	1.731E–11	30	3.3
10	1998/02/24	1998/03/25	1.287E–08	23	2.3
11	2010/03/18	2010/04/16	1.394E–07	11	0.3
12	2003/01/29	2003/02/20	3.500E–07	14	0.7
13	2013/07/01	2013/07/01	1.186E–06	5	<0.1
14	1998/10/21	1998/11/02	1.682E–06	12	0.5
15	2005/06/22	2005/07/13	2.679E–05	6	<0.1
16	2005/10/05	2005/10/18	7.139E–05	12	0.6
17	1997/06/16	1997/06/24	8.885E–05	4	<0.1
18	1999/06/10	1999/06/11	0.0001	4	<0.1
19	1998/04/29	1998/05/21	0.0002	10	0.4
20	2011/09/20	2011/10/19	0.0002	21	3.0
21	2013/06/18	2013/07/04	0.0003	10	0.4
22	2006/09/22	2006/09/25	0.0011	4	<0.1
23	2008/08/22	2008/09/17	0.0014	11	0.6
24	2000/11/20	2000/12/08	0.0022	7	0.1
25	2012/10/26	2012/10/29	0.0026	4	<0.1
26	2010/03/08	2010/03/22	0.0029	9	0.4
27	2007/08/16	2007/09/14	0.0037	10	0.5
28	2011/02/14	2011/03/10	0.0041	8	0.2
29	2007/11/21	2007/11/29	0.0058	5	<0.1
30	2005/11/08	2005/11/24	0.0068	5	<0.1
31	2008/11/14	2008/11/14	0.0110	3	<0.1
32	2015/08/19	2015/09/14	0.0130	13	1.2
33	2008/03/05	2008/03/14	0.0170	4	<0.1
34	2001/02/22	2001/02/23	0.0190	4	<0.1
35	2001/10/05	2001/10/26	0.0200	14	1.6
36	2001/04/26	2001/04/30	0.0270	6	0.1
37	1999/08/26	1999/08/26	0.0390	4	<0.1
38	2003/10/22	2003/11/18	0.0470	10	0.7

### Comparison to severe weather events

Around a third (34.2%, 13/38) of the 38 statistically significant cryptosporidiosis clusters showed temporal and spatial alignment with severe weather events from NIWA’s Catalogue (Table 2). There were approximately 236 severe weather events listed in the Catalogue for the study period (1997–2015), but only 13 of the 236 (5.5%) severe weather events showed alignment with significant cryptosporidiosis clusters. Several other clusters also showed temporal and spatial alignment with severe weather events but were excluded from further analysis because the weather events did not include heavy rainfall or because the cluster had a known cause unrelated to weather. The number of cases in the 13 clusters that aligned with severe weather events ranged from 3 to 55 (mean = 12.2, median = 10). Nearly half (46.2%, 6/13) of the 13 clusters that aligned with severe weather events occurred in the spring, three occurred in the winter, three occurred in the autumn, and one occurred in the late summer and early autumn (Table 2).

**Table 2.** Temporal alignment of statistically significant SaTScan-detected cryptosporidiosis clusters with severe weather events from NIWA’s Historic Weather Event Catalogue

Statistically significant SaTScan-detected clusters		Corresponding severe weather events					
Cluster number	3 average incubation periods (7 days x 3) before the start date of the cluster^a^	Name of event(s)^b^	Start date^a^	End date^a^	Season	Number of days between the start of severe weather event and the start of cluster	Number of days between the end of severe weather event and the start of cluster
2^c^	1999/03/02	March 1999 Kaikoura Rain	1999/03/12	1999/03/14	Autumn	11	9
6^c^	2000/10/04	(i) October 2000 New Zealand Weather Bomb	2000/10/11	2000/10/13	Spring	14	12
		(ii) October 2000 Manawatu–Whanganui and Wellington Flooding	2000/10/09	2000/10/11		16	14
11	2010/02/25	March 2010 Wellington and Marlborough Storm	2010/03/12	2010/03/12	Autumn	6	6
13^c^	2013/06/10	June 2013 New Zealand Storm	2013/06/20	2013/06/22	Winter	11	9
14	1998/09/30	October 1998 New Zealand High Winds and Flooding	1998/10/18	1998/10/21	Spring	3	0
16	2005/09/14	September 2005 North Island Winds and South Island Snow	2005/09/16	2005/09/19	Spring	19	16
17	1997/05/25	June 1997 Northland Flooding and Hawke’s Bay Flooding	1997/06/01	1997/06/03	Winter	15	13
27	2007/07/26	July–August 2007 New Zealand Storm	2007/07/29	2007/08/01	Winter	18	15
30	2005/10/18	October 2005 Gisborne and Hawke’s Bay Flooding	2005/10/20	2005/10/22	Spring	19	17
31^c^	2008/10/24	(i) November 2008 South Island Cold Snap	2008/11/04	2008/11/07	Spring	10	7
		(ii) November 2008 Wellington Wind	2008/11/01	2008/11/01		13	13
33	2008/02/13	(i) March 2008 New Zealand Storm	2008/02/29	2008/03/02	Summer/autumn	5	3
		(ii) February 2008 Upper North Island Storm	2008/02/22	2008/02/23		12	11
36^c^	2001/04/05	April 2001 North Island Ex–Tropical Cyclone Sose	2001/04/12	2001/04/14	Autumn	14	12
38	2003/10/01	October 2003 New Zealand Storm	2003/10/03	2003/10/05	Spring	19	17

*Note:* Clusters not listed in this table either (i) did not have temporal alignment with a weather event, (ii) did not have spatial alignment with a weather event, (iii) had a known cause unrelated to weather (e.g. direct exposure to infected calves at a university led to an outbreak among veterinary students), or (iv) there was no evidence of increased rainfall in NIWA’s Catalogue or in the total precipitation data for CAUs for the associated weather event(s).

a Dates are presented in yyyy/mm/dd format.

b Names of events were copied directly from NIWA’s Catalogue, but do not necessarily reflect the full extent of impacts.

c Associated with known outbreak (see Table 3 for more details).

**Figure 1.**
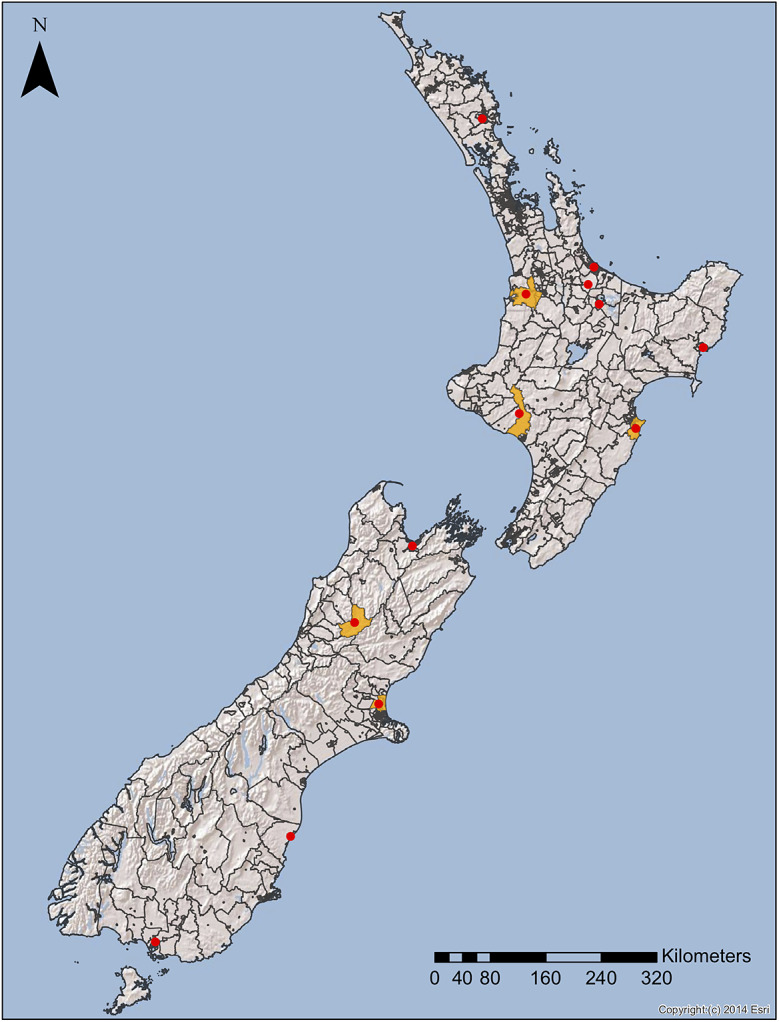
Space–time clusters of cryptosporidiosis in NZ (1997–2015) at the CAU level as identified by spatial scan statistic in SaTScan that align temporally and spatially with severe weather events. The orange regions (with red dots at the CAU centroids) are CAUs with statistically significant space–time clusters (*p* < 0.05).

Statistically significant clusters that align with severe weather events are indicated in Figure 1. The median time between the start of severe weather and the start of a significant cluster was 14 days (range = 3, 19).

While NIWA Catalogue entries for five of the 16 severe weather events that occurred before the detected cryptosporidiosis clusters mentioned ‘sewage’ or ‘sewerage’ issues and one mentioned a boil water notice, none of the reported issues overlapped with the locations of the clusters. However, the NIWA Catalogue does not systematically record such issues and relies on media reports for such details of storm impacts.

### Comparison with daily total precipitation and maximum temperature

Temporal patterns of daily total precipitation and maximum temperature prior to and during clusters were explored for the first CAU listed for each cluster. The mean highest total daily precipitation in the 21 days prior to the clusters was 24.92 mm (range = 6.06, 58.79). The mean lowest daily maximum temperature in the 21 days prior to the clusters was 283.16 K [10.01 °C] (range = 277.61, 288.01), while the mean highest daily maximum temperature in the 21 days prior to clusters was 291.01 K [17.86 °C] (range = 286.05, 295.81).

#### Cluster 2

There was a small increase in precipitation (circled in red, Supplementary Figure 1) in mid-March 1999 that aligned with the ‘March 1999 Kaikoura Rain’ severe weather event, which occurred approximately a week and a half before the start date of the cryptosporidiosis cluster.

#### Cluster 6

There was a slight increase in precipitation (circled in red, Supplementary Figure 2) in early October 2000 that aligned with the ‘October 2000 Manawatu-Whanganui and Wellington Flooding’ event, which directly preceded a large peak in total precipitation on 11 October (also circled in red) that aligned with the ‘October 2000 New Zealand Weather Bomb’ that occurred approximately two weeks before the cluster start date.

#### Cluster 11

There was a small increase in total precipitation on 12 March that aligned with the ‘March 2010 Wellington and Marlborough Storm’ that affected the top of the South Island six days before the start of the cluster (circled in red, Supplementary Figure 3). However, it should be noted that the cluster included six different CAUs and the total precipitation estimate is only for one of the CAUs. Therefore, it is possible that rainfall totals were higher in other areas.

#### Cluster 13

A period of precipitation (circled in red, Supplementary Figure 4) aligned with the ‘June 2013 New Zealand Storm’ that impacted the country ten days prior to the start of the detected cluster.

#### Cluster 14

A period of precipitation three days before the start of the cluster, circled in red in Supplementary Figure 5, aligned with the ‘October 1998 New Zealand High Winds and Flooding’ event.

#### Cluster 16

A small peak in total precipitation (circled in red, Supplementary Figure 6) aligned with the ‘September 2005 North Island Winds and South Island Snow’ event from 16 to 19 September. While the event primarily reported snow at higher elevations, it likely produced substantial rainfall at lower elevations as well.

#### Cluster 17

**Figure 2.**
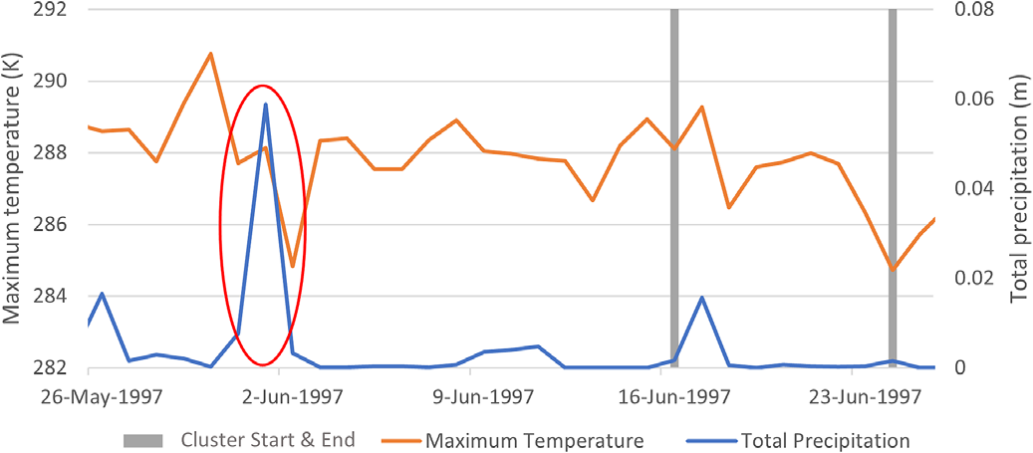
Patterns of daily total precipitation and maximum temperature in identifying CAU in the three weeks prior and for the duration of Cluster 17 (see Table 2).

The large peak in total precipitation on 1 June 1997 (circled in red, Figure 2) aligned with the ‘June 1997 Northland Flooding and Hawke’s Bay Flooding’ event, which occurred around two weeks prior to the start of the cluster.

#### Cluster 27

A small peak in total precipitation in late July and early August 2007 (circled in red, Supplementary Figure 7) aligned with the ‘July–August 2007 New Zealand Storm’ event, which occurred just over two weeks before the cluster start date. The storm event reportedly brought unsettled weather to the North Island.

#### Cluster 30

**Figure 3.**
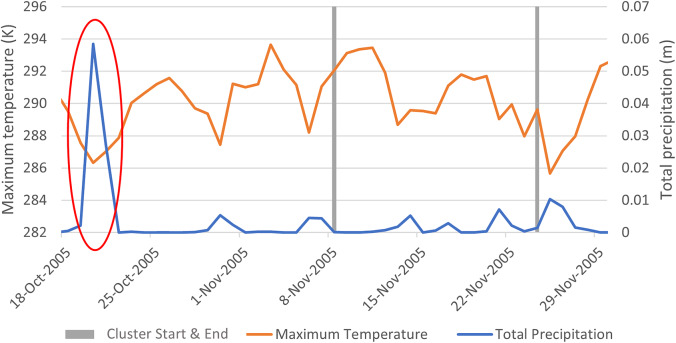
Patterns of daily total precipitation and maximum temperature in identifying CAU in the three weeks prior and for the duration of Cluster 30 (see Table 2).

The large peak in total precipitation (circled in red, Figure 3) aligned with the ‘October 2005 Gisborne and Hawke’s Bay Flooding’ event, which brought heavy rain to the Bay of Plenty region.

#### Cluster 31

A peak in daily total precipitation (circled in red, Supplementary Figure 8) aligned with the ‘November 2008 Wellington Wind’ event on 1 November 2008, which brought heavy rain to the West Coast, including Buller. Another later peak in total precipitation aligned with the ‘November 2008 South Island Cold Snap’ event from 4 to 7 November 2008, which brought both heavy rain and snow to the West Coast. Additional peaks in daily total precipitation were evident, but they did not coincide with any of the severe weather events included in NIWA’s Catalogue.

#### Cluster 33

A small peak in total precipitation in February (circled in red, Supplementary Figure 9) had a degree of alignment with the ‘February 2008 Upper North Island Storm’ event, which reportedly impacted the North Island from 22 to 23 February 2008. A large peak in precipitation in March also had a degree of alignment with the ‘March 2008 New Zealand Storm’ event, which impacted the country at the beginning of the month, in the week before the cluster start date.

#### Cluster 36

A large peak in total precipitation (circled in red, Supplementary Figure 10) aligned with the ‘April 2001 North Island Ex-Tropical Cyclone Sose’ event, which impacted many parts of the North Island from 12 to 14 April 2001. The storm arrived 12 days prior to the start of the detected cluster.

#### Cluster 38

A peak in total precipitation (circled in red, Supplementary Figure 11) aligned closely with the ‘October 2003 New Zealand Storm’ event, which brought heavy rain to much of the North Island from 3 to 5 October 2003. Another peak in daily total precipitation was observed approximately one week later, but the peak did not coincide with any of the severe weather events included in NIWA’s Catalogue.

### Comparison to previously recorded outbreaks

Five of the 13 statistically significant cryptosporidiosis clusters that showed alignment with severe weather events had at least one case with an assigned outbreak number (Table 3; see Supplementary Text for additional details).

**Table 3.** Statistically detected space–time clusters of cryptosporidiosis and temporally and spatially overlapping recorded outbreaks of cryptosporidiosis in New Zealand, 1997–2015

Significant SaTScan-detected clusters				Corresponding outbreak(s)			
Cluster number	Start date^a^	End date^a^	Observed cases	Corresponding outbreak #	Start date^a^	End date^a^	Total cases^b^
2	1999/03/02	1999/04/20	55	CB1999009	1999/02/26	1999/04/23	61
6	2000/10/25	2000/10/27	22	RO2000015	1999/12/09	—^c^	27
11	2010/03/18	2010/04/16	11	—	—	—	—
13	2013/07/01	2013/07/01	5	OB–13–104077–WG	2013/04/28	2013/06/20^d^	6
14	1998/10/21	1998/11/02	12	—	—	—	—
16	2005/10/05	2005/10/18	12	—	—	—	—
17	1997/06/16	1997/06/24	4	—	—	—	—
27	2007/08/16	2007/09/14	10	—	—	—	—
30	2005/11/08	2005/11/24	5	—	—	—	—
31	2008/11/14	2008/11/14	3	OB–08–100818–GM	2008/10/31	—^c^	4
33	2008/03/05	2008/03/14	4	—	—	—	—
36	2001/04/26	2001/04/30	6	—	—	—	—
38	2003/10/22	2003/11/18	10	RO2003011	2003/11/01	2003/11/12	6

a Dates are presented in yyyy/mm/dd format.

b Total cases include laboratory-confirmed, clinically confirmed, and probable cases.

c Outbreak end date was not reported, but the outbreak report date fell within the cluster date range.

d Outbreak end date did not fall within the cluster date range, but the report date for the outbreak fell within the cluster date range.

When the outbreaks were compared to the corresponding severe weather events, it became apparent that for four of the outbreaks (Table 3), the start date preceded the severe weather event start date (Table 2), while only one outbreak began after the severe weather event (outbreak number RO2003011; Table 3). However, severe weather events may lead to the further spread of an existing outbreak.

The other eight clusters did not match any cases that were associated with known outbreaks (Clusters 11, 14, 16, 17, 27, 30, 33, and 36; Table 3), which implies that statistically significant cryptosporidiosis outbreaks were undetected by the surveillance system during the period of this study.

## Discussion

### Main findings and interpretation

SaTScan analysis detected 38 statistically significant cryptosporidiosis space–time clusters. Approximately 4% of notified cryptosporidiosis cases from 1997 to 2015 were part of these clusters. By comparison, 9% of notified cases for the same period were associated with an outbreak that had been identified by routine surveillance. These findings suggest that most cryptosporidiosis cases in NZ are sporadic (i.e. cases with no statistically significant spatial and temporal relationship with other confirmed cases) and not associated with clusters (i.e. as operationally defined in this study as three or more confirmed cases with a spatial and temporal relationship). However, the space–time scan statistic was limited to a radius of 25 km in this study to indicate localized exposure. Therefore, it is possible that regional increases in cases (e.g. cases spread beyond 25 km) might not be considered a single cluster. For example, heavy rainfall could lead to a regional increase in cryptosporidiosis cases that are not linked to a single contaminated water supply, but rather to the contamination of multiple small water sources (e.g. bore wells) in the region.

Around a third (34.2%, 13/38) of the statistically significant cryptosporidiosis clusters showed temporal and spatial alignment with severe weather events from NIWA’s Catalogue. Of these, nearly half (46.2%, 6/13) occurred in the spring. Clusters detected during the spring months may be due to the transmission of the zoonotic *Cryptosporidium parvum (C. parvum),* rather than *Cryptosporidium hominis (C. hominis)*, as previous research has indicated that there are seasonal strain-specific transmission cycles, with zoonotic transmission in the spring and anthroponotic transmission in the autumn [23, 35]. Spring peaks in cryptosporidiosis in rural areas may be associated with spring calving and lambing because newborn livestock can be an important source of this infection [36–38]. Spring clusters of cryptosporidiosis in rural areas of NZ may also be associated with rainfall [4]. For example, a study in England showed that peaks in spring rainfall preceded peaks in cryptosporidiosis notifications [39] and local rainfall has been reported as a determinant of cryptosporidiosis infection in children globally [2]. Studies have also shown that heavy rainfall events can significantly increase surface runoff of cryptosporidium oocysts over land used for livestock agriculture [7–10]. Furthermore, heavy rainfall events have been associated with increased cryptosporidium pathogen loads in waterways and increased cryptosporidiosis incidence [11–13].

Only one of the statistically significant cryptosporidiosis clusters showing temporal and spatial alignment with severe weather events occurred in the summer (Cluster 33, Table 2). This suggests that recreational contact with contaminated waterways following heavy rainfall may be a less important transmission pathway than the consumption of contaminated drinking water in the development of cryptosporidiosis outbreaks. However, a clear pattern did not emerge when the daily maximum temperature was plotted for the 21 days prior to each cluster. Diffuse clusters or outbreaks may also involve other transmission routes. Person-to-person transmission may be particularly important with a number of cases linked to attendance at childcare centres or the changing of dirty diapers in NZ [36].

While 13 of the 38 (34.2%) significant cryptosporidiosis clusters showed alignment with severe weather events, it is important to note that only 13 of the 236 (5.5%) severe weather events showed alignment with significant cryptosporidiosis clusters. This finding indicates that only a small proportion of severe weather events may contribute to identifiable outbreaks of cryptosporidiosis. However, it would be beneficial to compare severe weather events to waterborne outbreaks of other gastrointestinal diseases (e.g. campylobacteriosis, giardiasis) to better ascertain the overall impact of such events on enteric disease outbreaks in NZ. Furthermore, the analysis of more recent disease data, especially following recent major weather events, would also be insightful.

When the daily total precipitation and maximum temperature were plotted for the identifying CAU for each cluster, it was clear that all the corresponding severe weather events caused increases in precipitation. However, for several clusters (Clusters 11, 14, and 16), the increase in precipitation in the identifying CAU was less than 10 mm per day. Cryptosporidiosis has been positively associated with rainfall in NZ [4, 5], but evidence for the relationship between cryptosporidiosis and temperature has been mixed [4-6]. Temperature, precipitation, and humidity affect the life cycle of *Cryptosporidium* spp. directly, as well as indirectly through ecological changes [1, 40].

Waterborne or environmental transmission was reported for five outbreaks identified by routine surveillance (corresponding with Clusters 2, 6, 13, 31, and 38; Table 3). The start date for four of these (corresponding with Clusters 2, 6, 13, and 31; Table 3) preceded the start of the corresponding severe weather event. This timing indicates that the corresponding severe weather events (Table 2) cannot have initiated those outbreaks (or the clusters that they closely align with). However, it is possible that severe weather contributed to the spread of infection via faecal–oral transmission.

### Strengths of this study

This is one of the first studies to detect space–time clusters of cryptosporidiosis in NZ. Lal et al. (2015) reported recurrent clusters of sporadic cryptosporidiosis cases for three time periods in NZ: 1997–2001, 2001–2004, and 2005–2008, while our study examined clusters over a longer time period (1997–2015). This is also the first study (to our knowledge) to compare detected clusters of cryptosporidiosis to severe weather events in NZ. Using SaTScan statistics in tandem with geographic information systems (GIS) techniques is useful for visualizing spatiotemporal patterns of disease risk [23, 41]. While some of the clusters detected in this study may have represented previously identified outbreaks, the space–time clusters highlight potential spatial and temporal variation in the drivers of disease.

### Limitations of this study

An important limitation of SaTScan is that when it is used over longer time periods, clusters may be detected because of underlying population changes in certain areas. The space–time scan statistic assumes that any change in the population at risk occurs uniformly across the study population [42], but demographic shifts were not considered in this study. Future studies may wish to consider the use of a discrete Poisson model, which would account for the underlying population at risk, or limit cluster detection to a single year at a time to prevent the risk of population shift bias. However, the benefit of using the space–time scan statistic in this study was that the model automatically adjusted for both purely spatial and purely temporal clusters (e.g. it accounted for seasonal patterns of disease).

Another potential limitation is the a priori choice of cluster size, but there are no clear guidelines for addressing this issue [42]. To address this potential limitation, we tested the space–time model using several different cluster sizes (e.g. varying maximum spatial radii and varying maximum temporal periods) before settling on the final parameters (i.e. 25 km maximum spatial radius and a 30-day temporal maximum). Additionally, it is possible that the use of a circular cluster window may result in the analysis missing non-circular clusters (e.g. those along a river) [42], although some studies have indicated that a circular cluster window can still successfully detect non-circular clusters of disease [43].

There are also limitations associated with the use of routine surveillance data to assess disease distribution, given the low-case ascertainment of enteric infections [23, 41] and errors in the geographic allocation of some that are identified [44]. In NZ, disease notifications report a case’s location based on the person’s home address. However, a person’s home location may not be an accurate reflection of the location of the source of the infection or the location of the exposure due to commuting or travel patterns to work or recreational settings. However, this error is unlikely to impact the detection of space–time clusters because they must exhibit both spatial variation and temporal variation [26]. It is also possible that the use of the onset date instead of the report date for notified cases could have improved the detection of disease clusters, as the onset date should be closer to the actual time of infection. However, onset dates were not available for all notified cases; therefore, the report dates for notifications were used in this study.

Another limitation was a change in the inclusion criteria for NIWA’s Catalogue in the 2010s [45]. This change may have led to the exclusion of some severe weather events that previously would have been included in the Catalogue prior to this decade. Additionally, the Catalogue generally excludes high rainfall events that have not been defined as storms or floods, but which may still have human health consequences. For example, a heavy rainfall event is thought to have led to the very large 2016 Havelock North campylobacteriosis outbreak [46], but this weather event was not recorded in NIWA’s Catalogue. Details on severe weather events in NIWA’s Catalogue are also limited. The Catalogue relies on newspaper and other media articles, which may selectively report examples of impacts. Therefore, the Catalogue may not capture impacts across all regions affected by the event. Furthermore, daily total precipitation and maximum temperature were only examined for the identifying CAU (i.e. the first CAU listed by SaTScan) for each cluster, but several detected clusters included multiple CAUs. Therefore, the plots may not be representative of conditions across the whole cluster area.

### Research and policy implications

This study found that heavy rainfall and flooding events preceded several detected cryptosporidiosis clusters in NZ from 1997 to 2015. However, additional research is required to determine the contribution of extreme weather events to cryptosporidiosis incidence and similarly for other enteric diseases (e.g. campylobacteriosis, giardiasis) in NZ. There is a pressing need for such research as rainfall intensity is projected to keep rising in NZ due to climate change [47, 48]. Similar research should also be conducted in other countries or regions with a high level of reliance on non-reticulated water supplies to assess the likelihood of enteric disease outbreaks associated with severe weather events.

The results suggest that a number of potential cryptosporidiosis outbreaks may have gone undetected in NZ. The epidemiological significance of SaTScan-detected clusters that have no evidence of a relationship to a known outbreak also merits further investigation. Enhancement of the disease surveillance system using SaTScan or a similar cluster detection tool, in combination with field investigations and typing, could help better identify and determine the cause of outbreaks. As noted above, clusters detected during the autumn months may be due to the transmission of *C. hominis* rather than *C. parvum*, because of seasonal strain-specific transmission cycles. Strain-specific data were not available for this study. The typing of all cases and outbreaks could help refine the disease surveillance system, as *C. hominis* and *C. parvum* have different drivers and risk factors.

This study supports the need to protect drinking water supplies from microbial contamination, especially from runoff from land used for livestock agriculture. Notifications and outbreaks of cryptosporidiosis, as well as other enteric diseases, have been linked to contaminated water supplies [14–16]. For example, the 2016 waterborne Havelock North campylobacteriosis outbreak was attributed to the contamination of two untreated bore wells with sheep faeces and resulted in approximately 7,570 cases and four deaths [46].

Such outbreaks highlight the need to strengthen the regulation and management of drinking water supplies to protect public health. Around 4,077,000 individuals in NZ (82.9% of the population) are served by public drinking water supply systems, but approximately 868,000 of those individuals (23.1%) receive drinking water that does not fully meet protozoal standards [49]. Furthermore, approximately 840,000 people in NZ (17.1% of the population) rely on non-reticulated water supplies [49], which may be particularly vulnerable to contamination. Action in this area is highly consistent with the principles of drinking water safety for NZ articulated by the Government Inquiry into Havelock North Drinking Water [50]. These principles include the paramount importance of protecting source water; the need for multiple barriers against contamination; that change (such as heavy rainfall or flooding) precedes contamination; and the need to apply a preventive risk management approach. This inquiry initiated the largest overhaul of drinking water supply in NZ’s history and the creation of a new national water regulator, Taumata Arowai. It will be important for this regulator to consider these principles and the findings of this current study as part of its mission to protect and improve drinking water quality.

## Conclusions

This study provides additional evidence that extreme weather events can play a role in the transmission of cryptosporidiosis in NZ. Many of the clusters that aligned temporally and spatially with severe weather events occurred in the spring and may be due to the transmission of the zoonotic *C. parvum.* Additional research is needed to assess the contribution of heavy rainfall and other environmental factors, such as livestock farming, to cryptosporidiosis cases and outbreaks, especially as rainfall intensity is projected to increase due to climate change.

## Supporting information

Grout et al. supplementary material Grout et al. supplementary material

## Data Availability

The disease notification data are available from ESR, but restrictions apply to the availability of these data, so they are not publicly available. Other data sets generated and analysed during this study are available from the corresponding author upon reasonable request.

## List of abbreviations

CAU: census area unit
ESR: Institute of Environmental Science and Research
NIWA: National Institute of Water and Atmospheric Research
NZ: New Zealand

## Supplementary material

For supplementary material accompanying this paper visit http://doi.org/10.1017/S095026882400058X. click here to view supplementary material
