# Supplementary material for: Severe weather events and cryptosporidiosis in Aotearoa New Zealand: A case series of space–time clusters
Source: Epidemiol Infect. 2024 Apr 15;152:e64. doi: 10.1017/S095026882400058X (PMC11062783; doi:10.1017/S095026882400058X)
Supplement: Grout et al. supplementary material [file S095026882400058Xsup001.zip › 6 Jan 23 - Severe weather and crypto clusters_Supp.docx]

**Epidemiology and Infection**

**Severe weather events and cryptosporidiosis in Aotearoa New Zealand: A case series of space-time clusters**

Leah Grout, Simon Hales, Michael G. Baker, Nigel French, Nick Wilson

**Supplemental Materials**

***Additional results for statistically significant space-time clusters associated with documented severe weather events***

**Supplementary Figure 1. Patterns of daily total precipitation and maximum temperature in the identifying CAU in the three weeks prior and for the duration of Cluster 2 (see Table 2).**

**Supplementary Figure 2. Patterns of daily total precipitation and maximum temperature in identifying CAU in the three weeks prior and for the duration of Cluster 6 (see Table 2).**

**Supplementary Figure 3. Patterns of daily total precipitation and maximum temperature in identifying CAU in the three weeks prior and for the duration of Cluster 11 (see Table 2).**

**Supplementary Figure 4. Patterns of daily total precipitation and maximum temperature in identifying CAU in the three weeks prior and for the duration of Cluster 13 (see Table 2).**

**Supplementary Figure 5. Patterns of daily total precipitation and maximum temperature in identifying CAU in the three weeks prior and for the duration of Cluster 14 (see Table 2).**

**Supplementary Figure 6. Patterns of daily total precipitation and maximum temperature in identifying CAU in the three weeks prior and for the duration of Cluster 16 (see Table 2).**

**Supplementary Figure 7. Patterns of daily total precipitation and maximum temperature in identifying CAU in the three weeks prior and for the duration of Cluster 27 (see Table 2).**

**Supplementary Figure 8. Patterns of daily total precipitation and maximum temperature in identifying CAU in the three weeks prior and for the duration of Cluster 31 (see Table 2).**

**Supplementary Figure 9. Patterns of daily total precipitation and maximum temperature in identifying CAU in the three weeks prior and for the duration of Cluster 33 (see Table 2).**

**Supplementary Figure 10. Patterns of daily total precipitation and maximum temperature in identifying CAU in the three weeks prior and for the duration of Cluster 36 (see Table 2).**

**Supplementary Figure 11. Patterns of daily total precipitation and maximum temperature in identifying CAU in the three weeks prior and for the duration of Cluster 38 (see Table 2).**

**Supplementary Text. Comparison to previously recorded outbreaks: Additional details**

A cluster in the Canterbury region from 23 March to 20 April 1999 included 55 notified cases (Cluster 2, Table 3) and closely matched an outbreak with 61 reported cases from 26 February to 23 April 1999. The outbreak was linked to a contaminated community swimming pool.

A cluster in the Bay of Plenty region from 25 October to 27 October 2000 (Cluster 6, Table 3) included 22 notified cases and had a degree of overlap with an outbreak with 27 reported cases that began on 9 December 1999. While the dates do not appear to match, no end date for the outbreak was recorded and the outbreak report was made on 25 October 2000. Therefore, it is possible that there was some overlap between the detected cluster and the reported outbreak if the reported outbreak occurred over a prolonged period. A number of cases from the outbreak were linked to a contaminated water supply on a farm.

Another cluster detected in the Bay of Plenty from 22 October to 18 November 2003 included 10 notified cases (Cluster 38, Table 3). The cluster partially matched an outbreak that had six cases which were reported from 1-12 November and was linked to a childcare center visit to a contaminated stream, where a group went swimming.

Another cluster detected in the Manawatu-Wanganui region on 1 July 2013 included five notified cases (Cluster 13, Table 3). The cluster may match an outbreak that reported six cases from 28 April to 20 June 2013. While the dates don’t closely align, the outbreak was reported on 1 July 2013 and the date used for the notified cases included in the cluster analysis was the report date for the notification rather than the onset date, which may explain the difference in dates. The outbreak was linked to a farm where cases had contact with dairy cattle. In addition, tests revealed that the water source had a low level of contamination.

A cluster detected in the West Coast region on 14 November 2008 included three cases (Cluster 31, Table 3) and displayed a degree of agreement with an outbreak that had four reported cases between 31 October and 14 November, when the outbreak report was submitted. The outbreak may have been linked to the consumption of untreated drinking water, poor hygiene, or contact with animals.
