# Supplementary figures and images for: Severe weather events and cryptosporidiosis in Aotearoa New Zealand: A case series of space–time clusters
Source: Epidemiol Infect. 2024 Apr 15;152:e64. doi: 10.1017/S095026882400058X (PMC11062783; doi:10.1017/S095026882400058X)

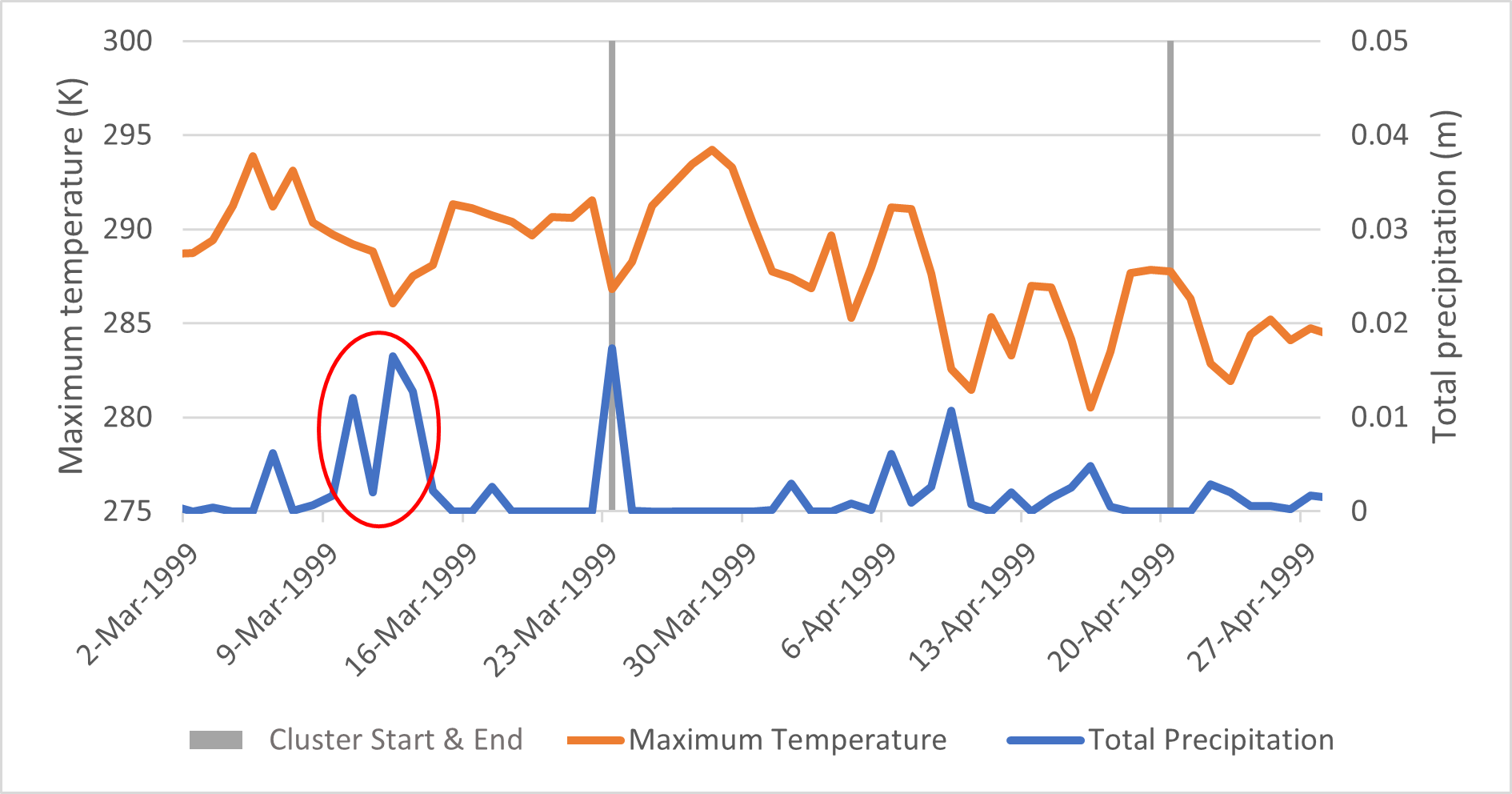

Supplement: Grout et al. supplementary material [file S095026882400058Xsup001.zip › Grout_EpiInfect_SuppFig1.png]

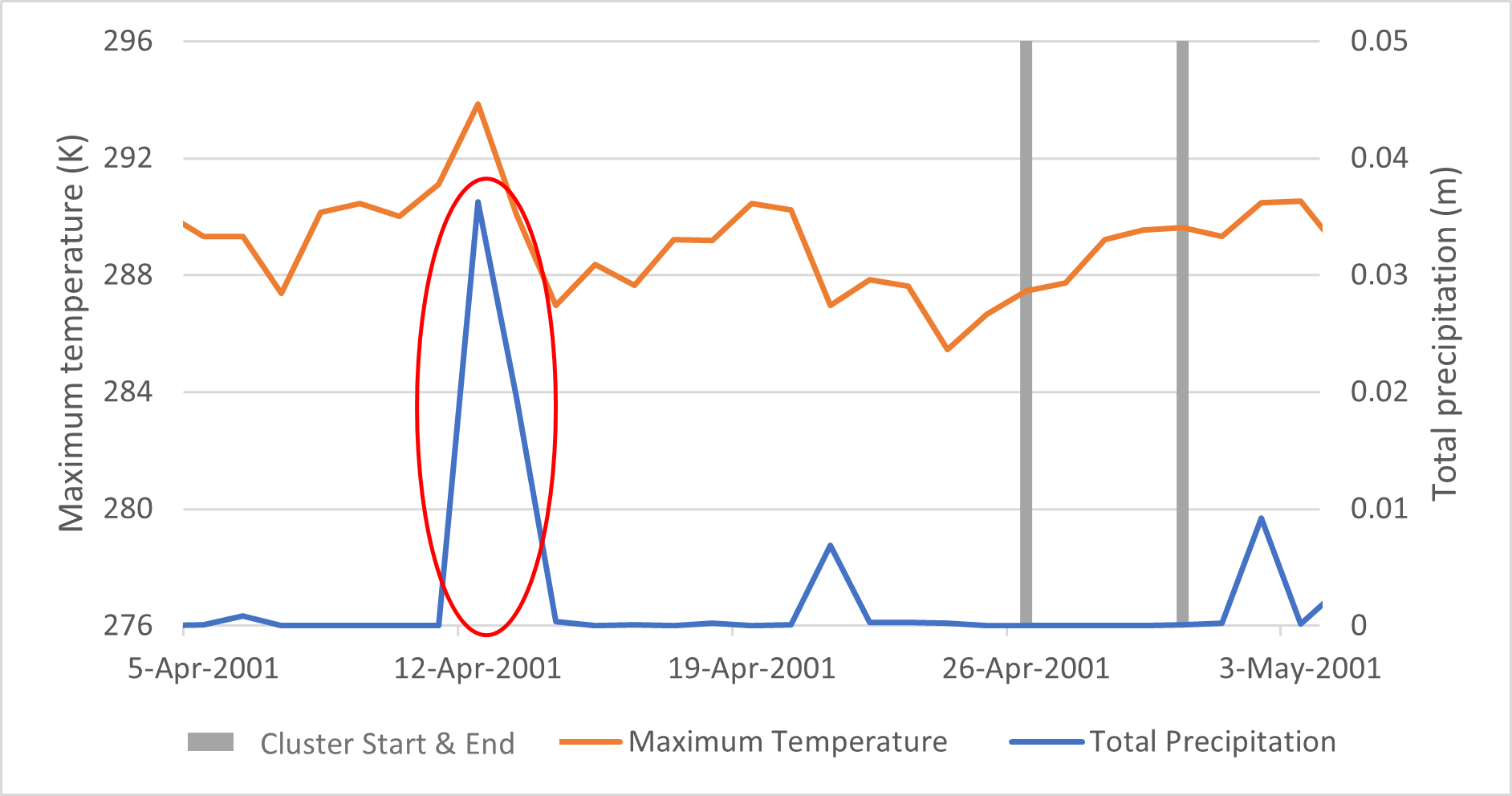

Supplement: Grout et al. supplementary material [file S095026882400058Xsup001.zip › Grout_EpiInfect_SuppFig10.png]

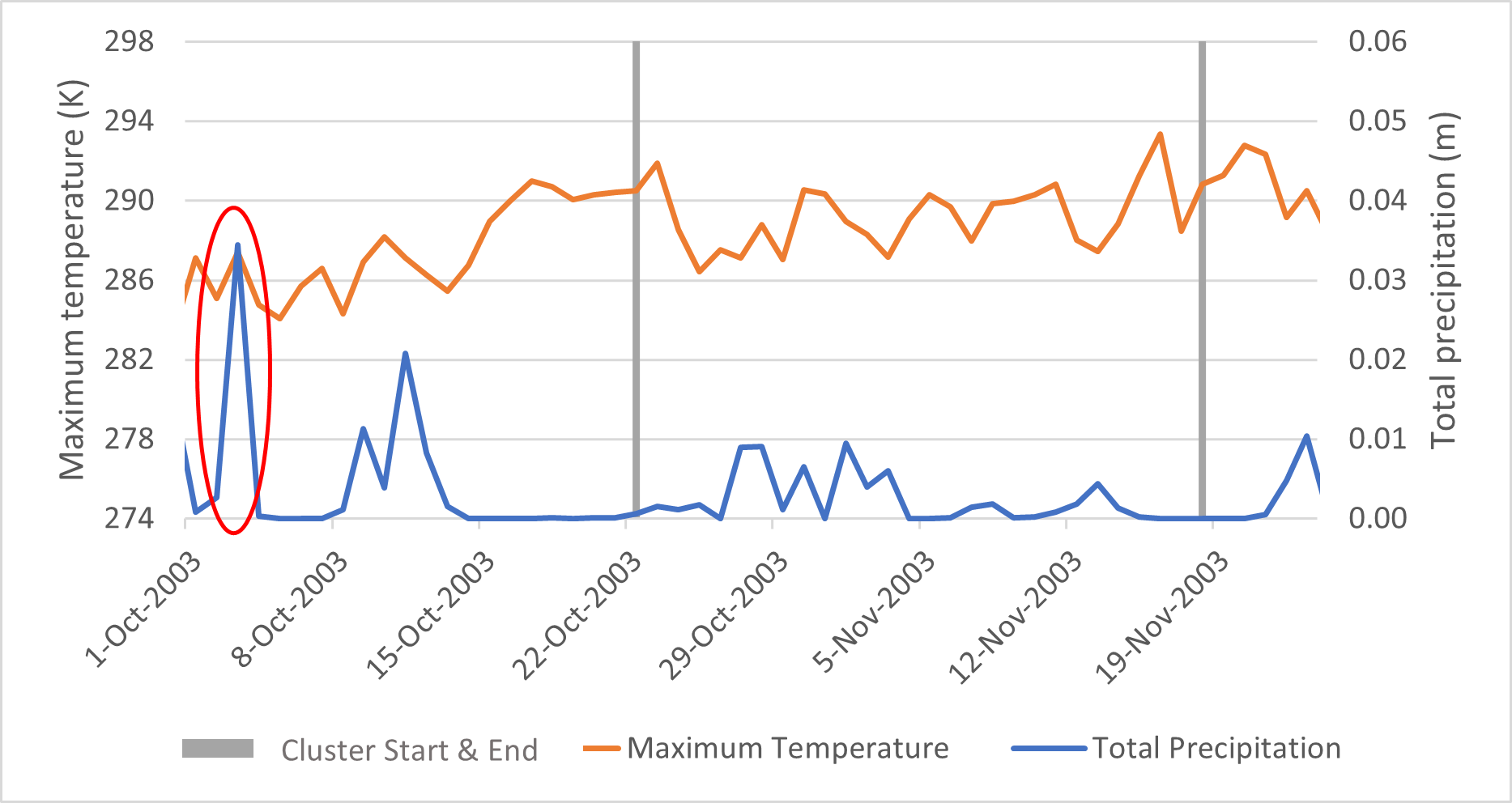

Supplement: Grout et al. supplementary material [file S095026882400058Xsup001.zip › Grout_EpiInfect_SuppFig11.png]

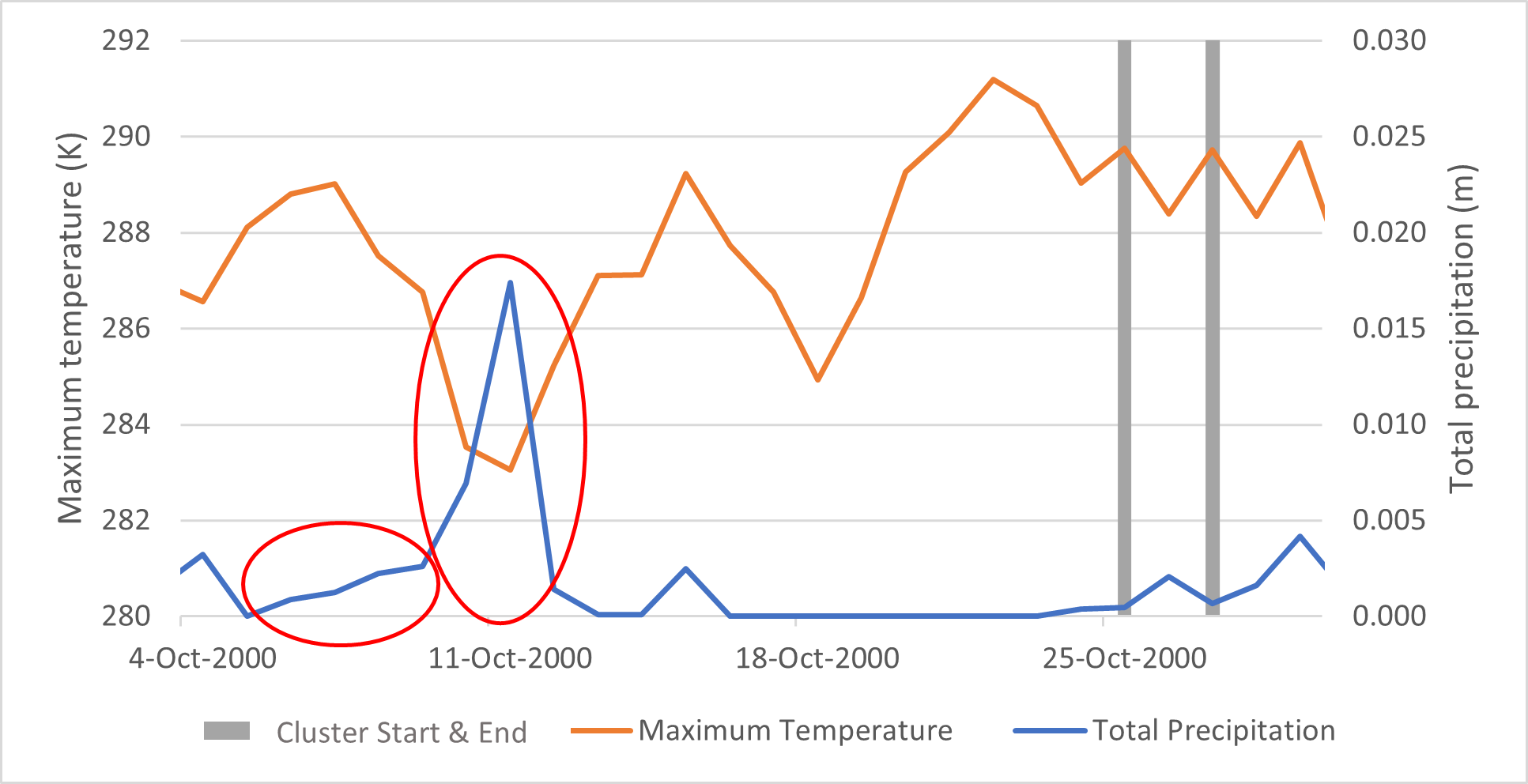

Supplement: Grout et al. supplementary material [file S095026882400058Xsup001.zip › Grout_EpiInfect_SuppFig2.png]

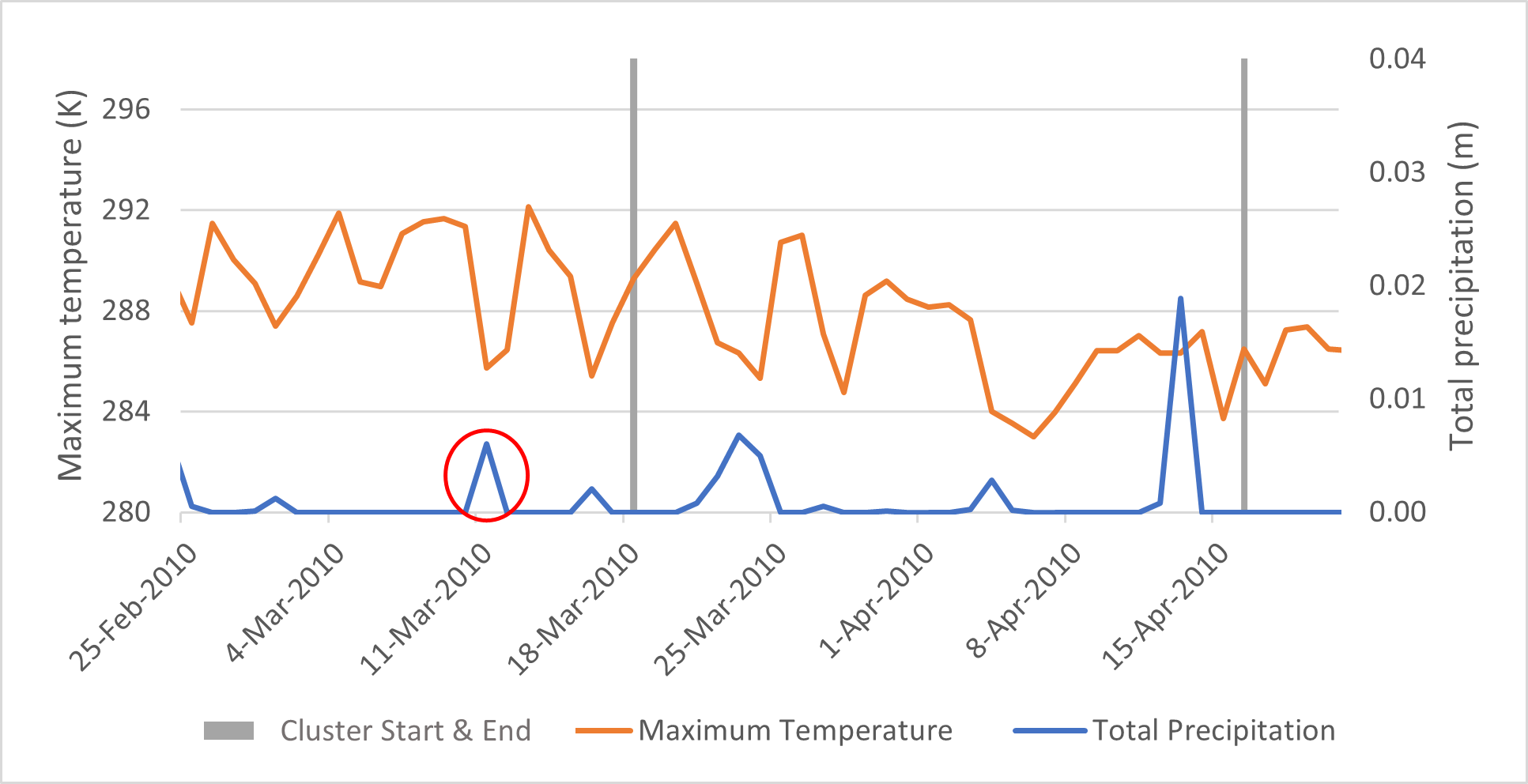

Supplement: Grout et al. supplementary material [file S095026882400058Xsup001.zip › Grout_EpiInfect_SuppFig3.png]

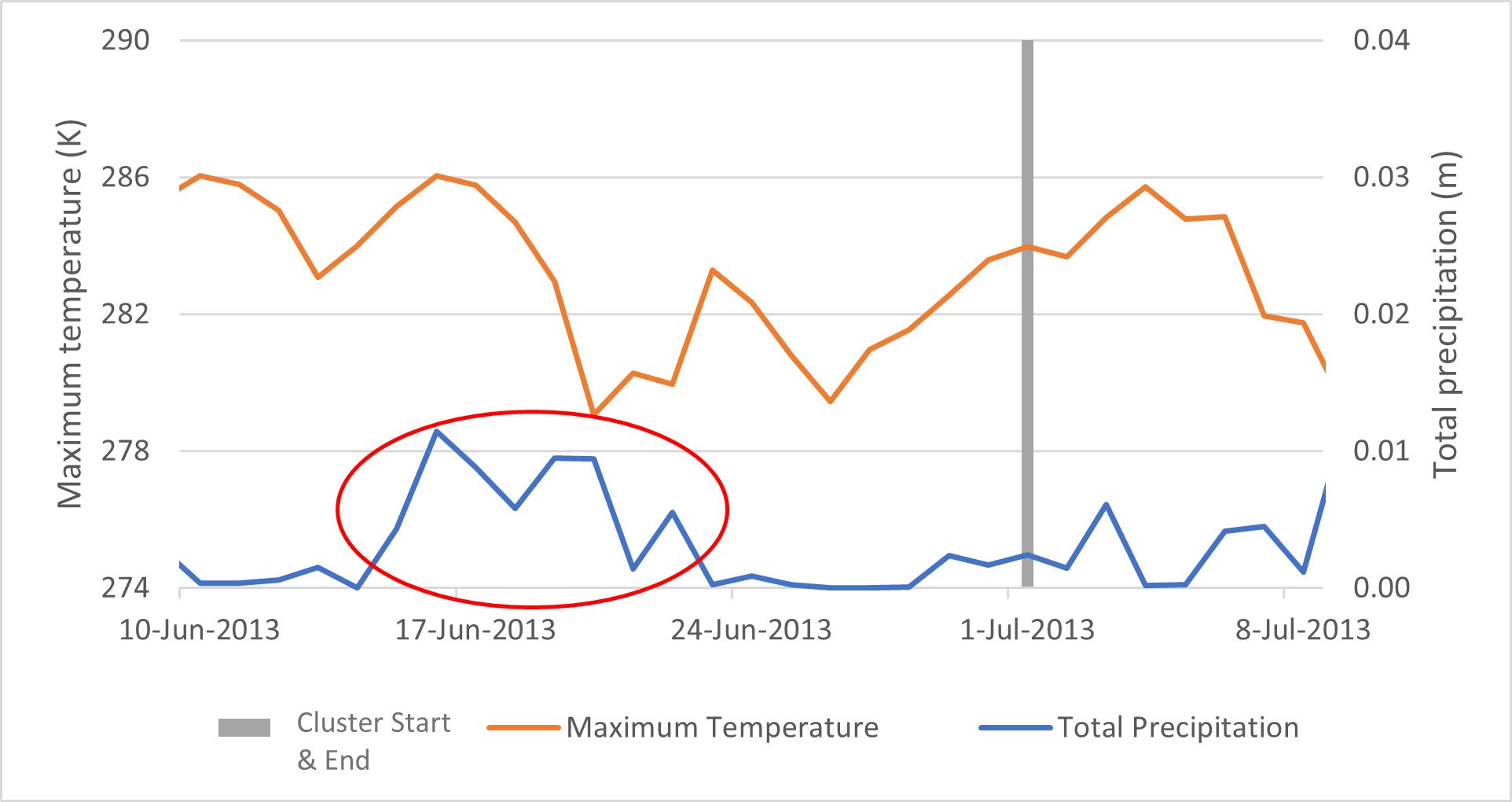

Supplement: Grout et al. supplementary material [file S095026882400058Xsup001.zip › Grout_EpiInfect_SuppFig4.png]

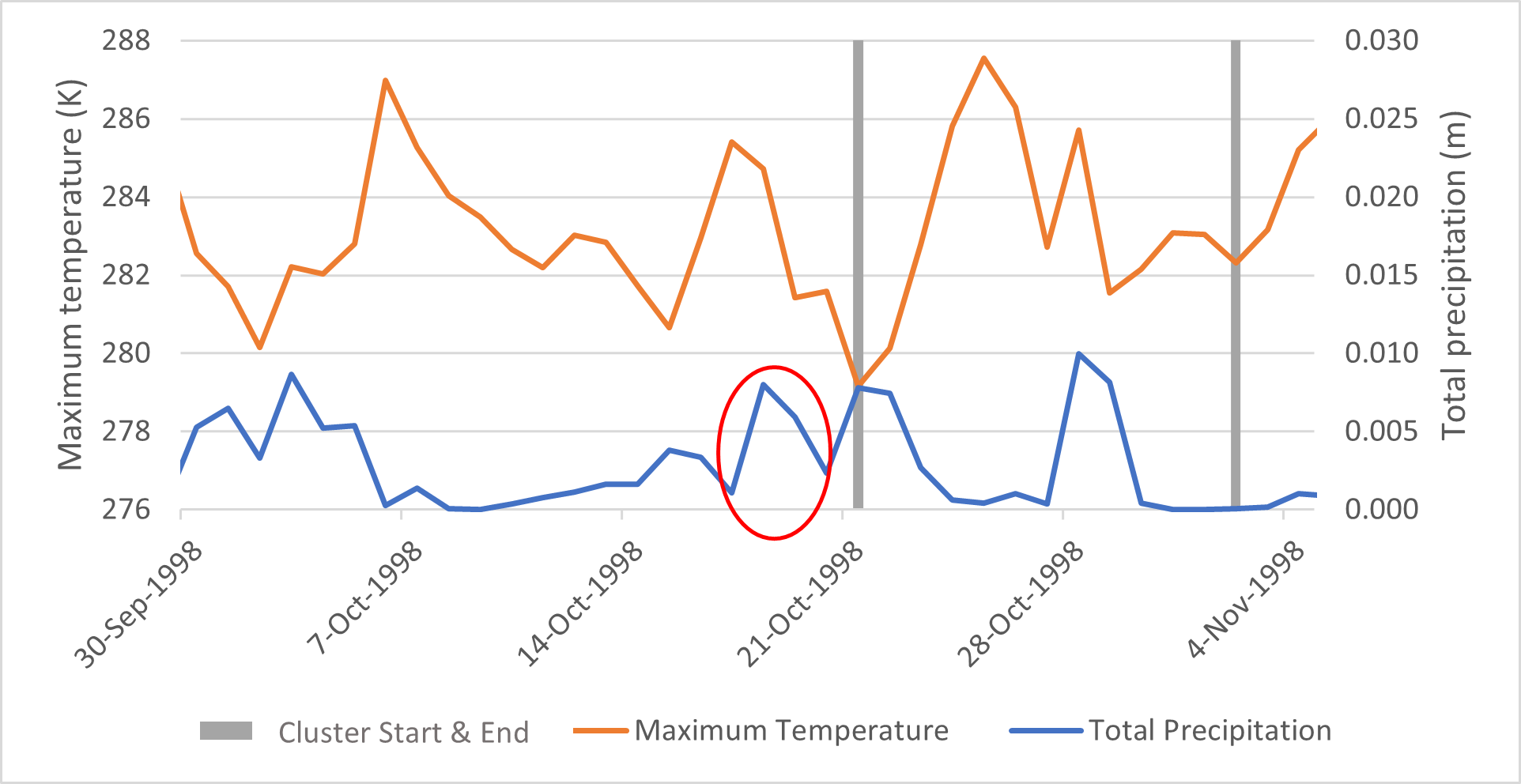

Supplement: Grout et al. supplementary material [file S095026882400058Xsup001.zip › Grout_EpiInfect_SuppFig5.png]

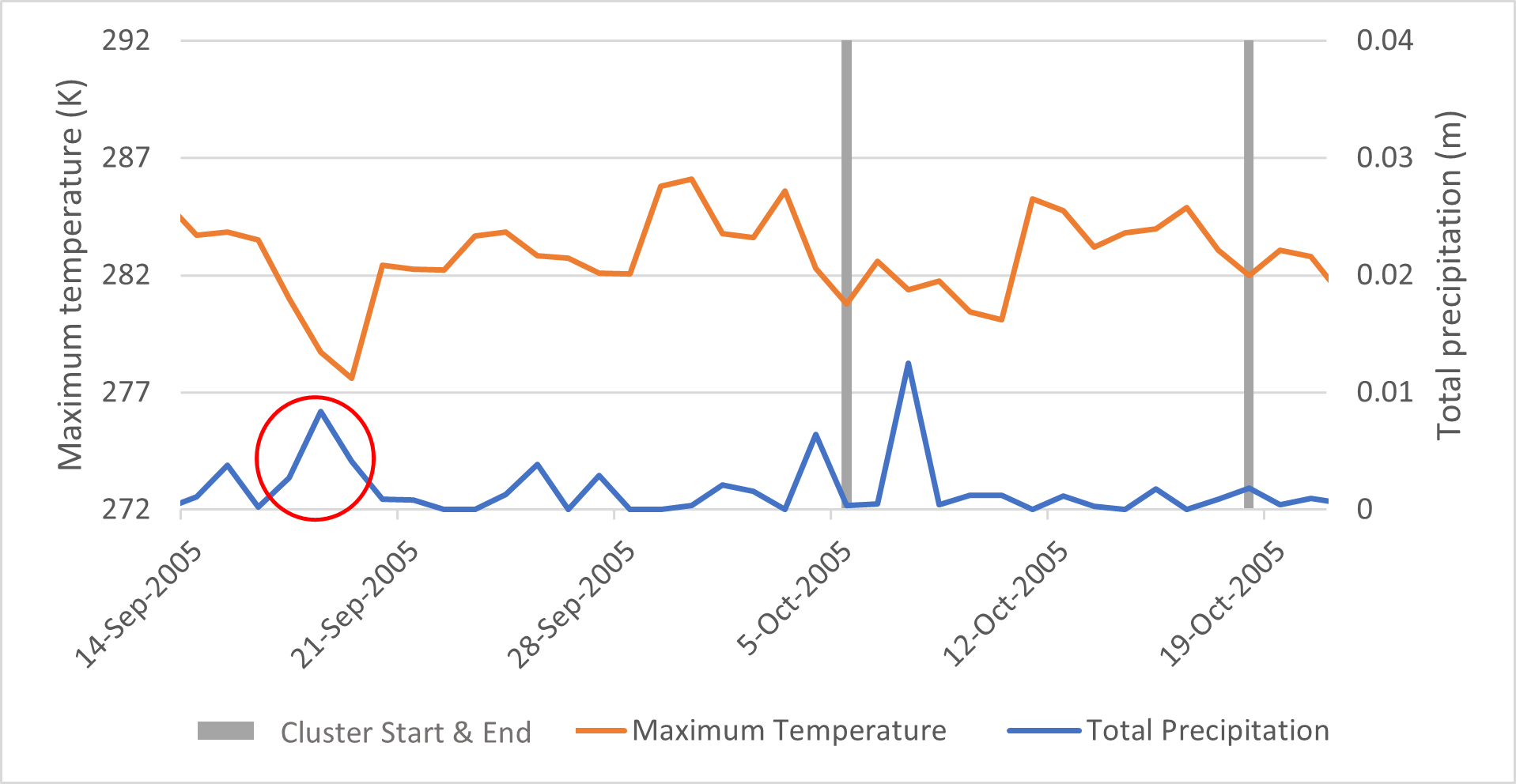

Supplement: Grout et al. supplementary material [file S095026882400058Xsup001.zip › Grout_EpiInfect_SuppFig6.png]

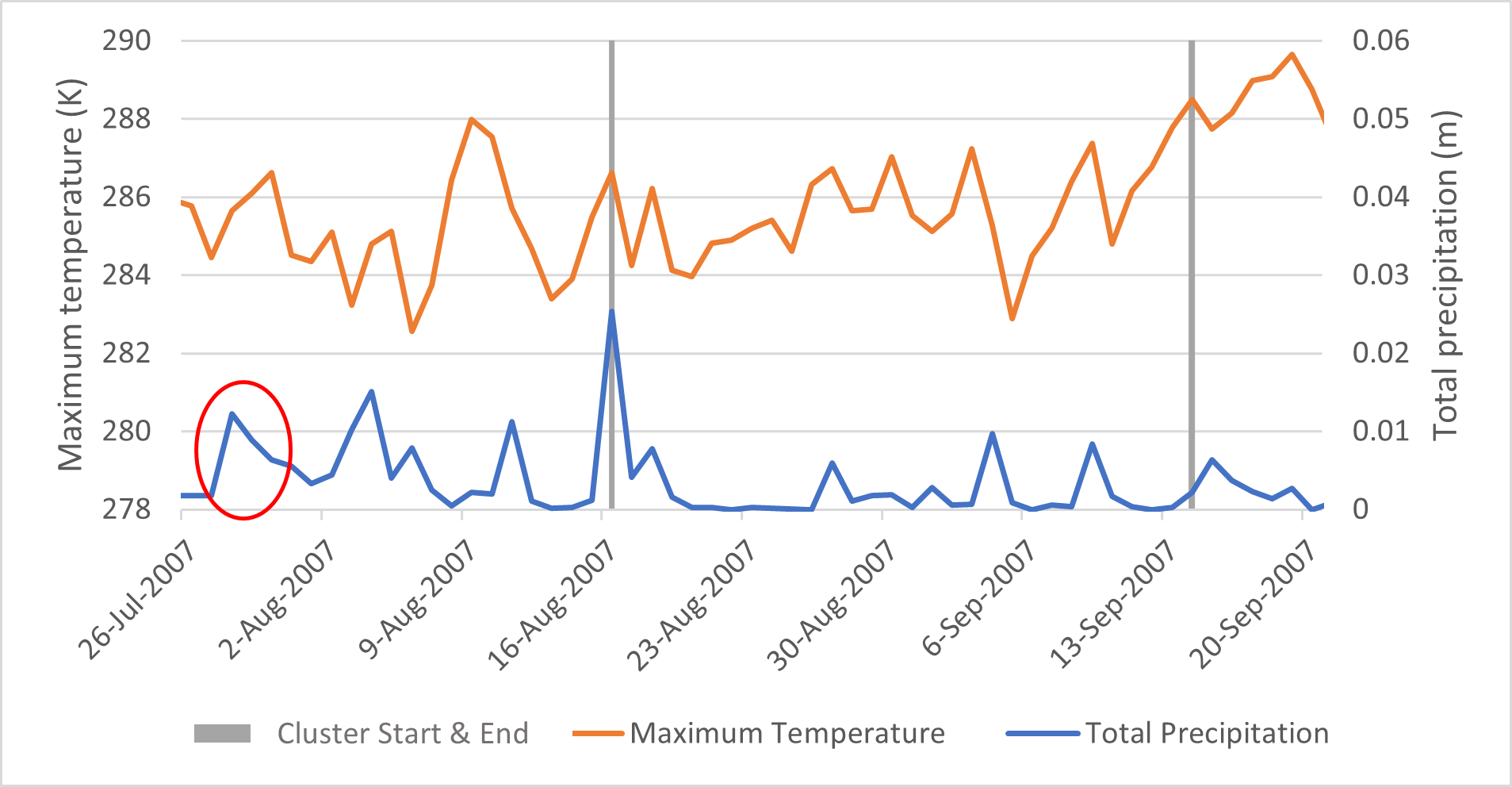

Supplement: Grout et al. supplementary material [file S095026882400058Xsup001.zip › Grout_EpiInfect_SuppFig7.png]

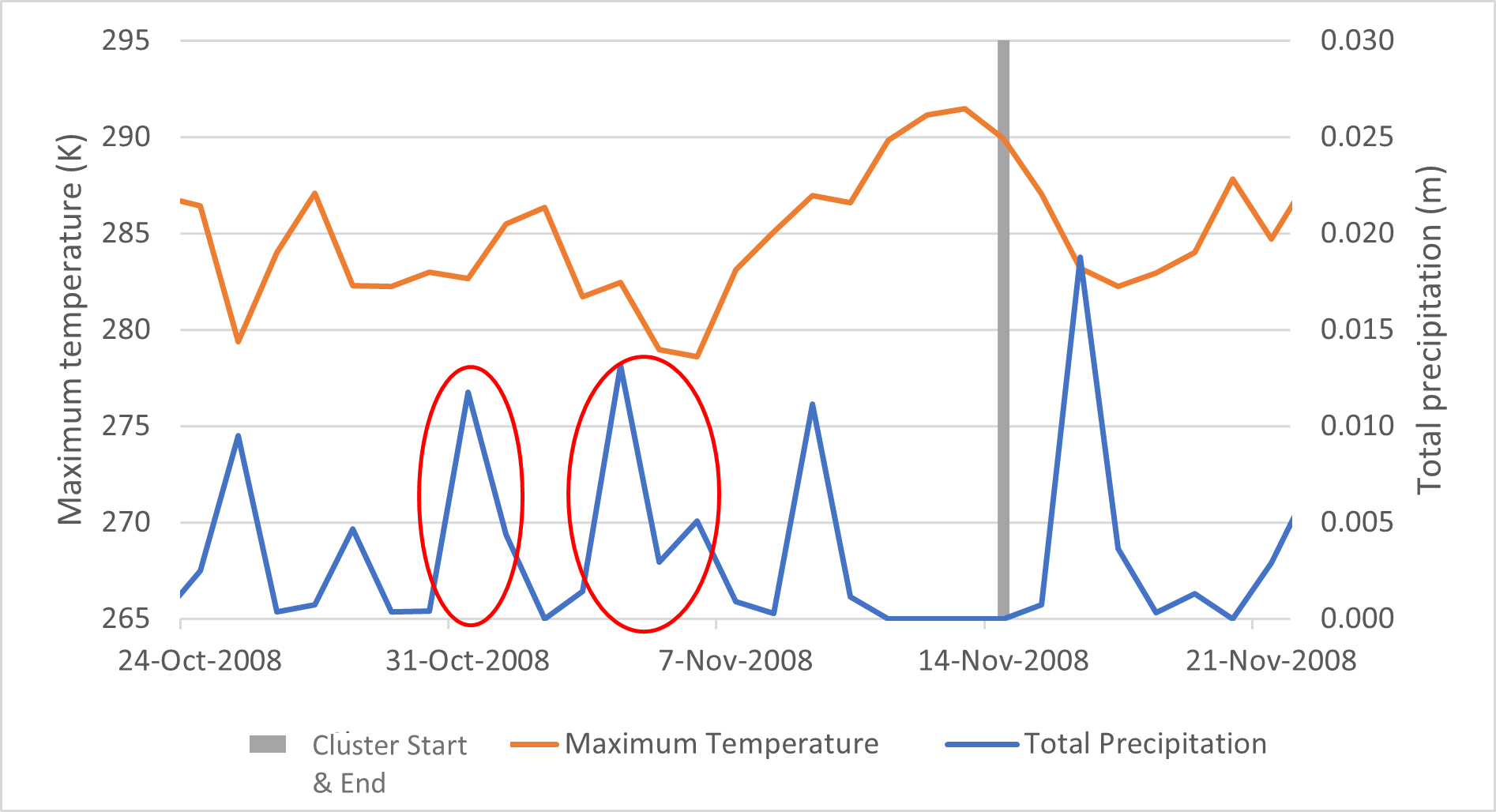

Supplement: Grout et al. supplementary material [file S095026882400058Xsup001.zip › Grout_EpiInfect_SuppFig8.png]

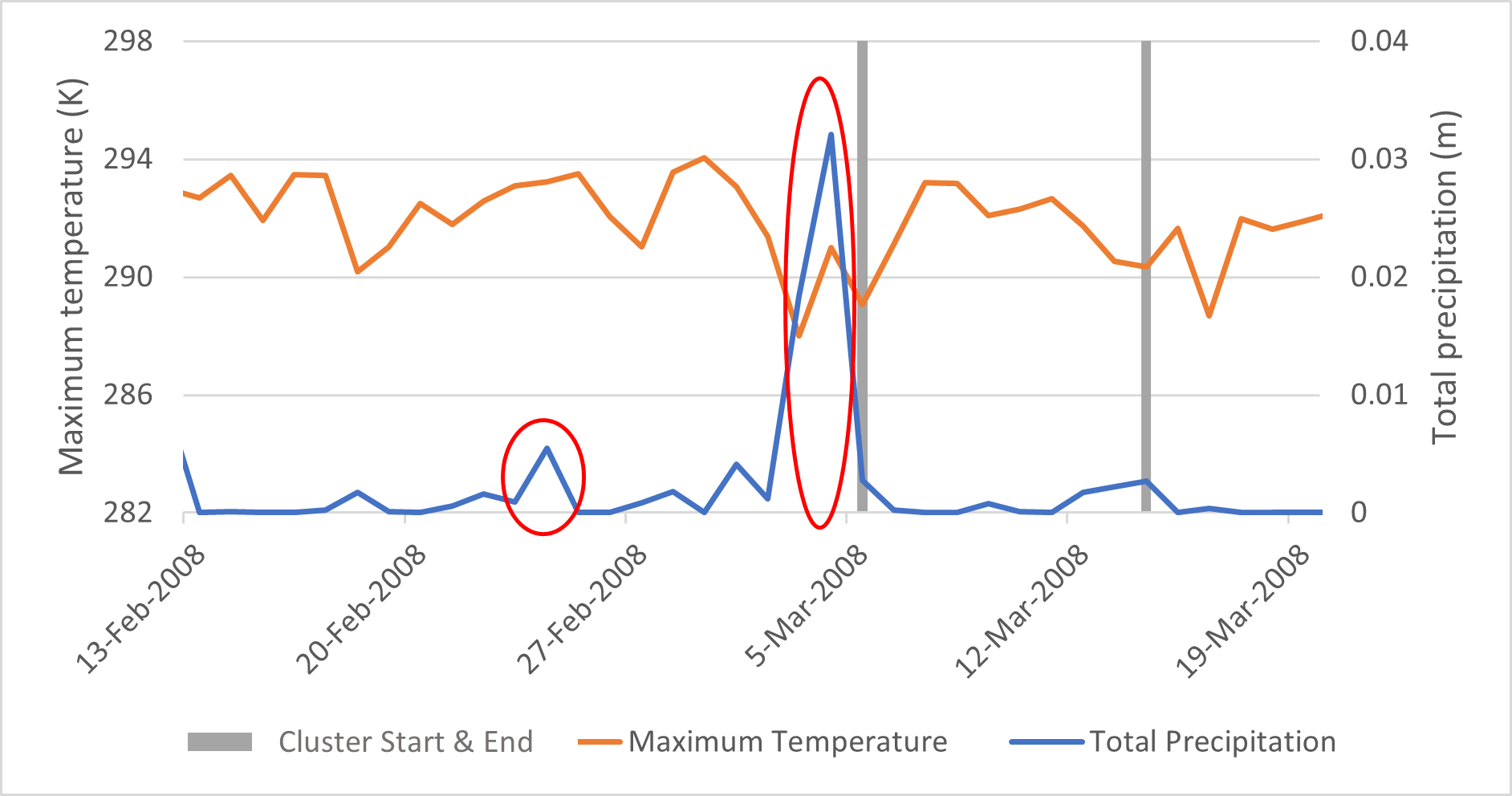

Supplement: Grout et al. supplementary material [file S095026882400058Xsup001.zip › Grout_EpiInfect_SuppFig9.png]
